# A Device-on-Chip Solution for Real-Time Diffuse Correlation Spectroscopy Using FPGA

**DOI:** 10.3390/bios14080384

**Published:** 2024-08-08

**Authors:** Christopher H. Moore, Ulas Sunar, Wei Lin

**Affiliations:** Department of Biomedical Engineering, Stony Brook University, Stony Brook, NY 11794, USA; christopher.h.moore@stonybrook.edu (C.H.M.); ulas.sunar@stonybrook.edu (U.S.)

**Keywords:** diffuse correlation spectroscopy, FPGA, device-on-chip, FPGA correlator

## Abstract

Diffuse correlation spectroscopy (DCS) is a non-invasive technology for the evaluation of blood perfusion in deep tissue. However, it requires high computational resources for data analysis, which poses challenges in its implementation for real-time applications. To address the unmet need, we developed a novel device-on-chip solution that fully integrates all the necessary computational components needed for DCS. It takes the output of a photon detector and determines the blood flow index (BFI). It is implemented on a field-programmable gate array (FPGA) chip including a multi-tau correlator for the calculation of the temporal light intensity autocorrelation function and a DCS analyzer to perform the curve fitting operation that derives the BFI at a rate of 6000 BFIs/s. The FPGA DCS system was evaluated against a lab-standard DCS system for both phantom and cuff ischemia studies. The results indicate that the autocorrelation of the light correlation and BFI from both the FPGA DCS and the reference DCS matched well. Furthermore, the FPGA DCS system was able to achieve a measurement rate of 50 Hz and resolve pulsatile blood flow. This can significantly lower the cost and footprint of the computational components of DCS and pave the way for portable, real-time DCS systems.

## 1. Introduction

Diffuse correlation spectroscopy (DCS) is an optical technique for non-invasively monitoring blood perfusion in microvasculature using the blood flow index (BFI) derived from the autocorrelation model of scattered light from moving blood cells, as detailed in previous reviews [1,2,3,4,5]. It has previously been shown that DCS measurements correlate well with established modalities such as arterial-spin labeled magnetic resonance imaging (ASL-MRI) [6,7,8] and Doppler ultrasound [3,7,9,10,11,12,13], with its advantages that DCS has a lower cost and more portability than MRI and more sensitivity to microvasculature than Doppler ultrasound. Thus, DCS has found numerous applications related to the bedside monitoring of several disease conditions including acute brain injuries [1,3,14,15,16,17,18,19,20,21,22,23,24] and the monitoring of cancer therapies [1,2,3,4,5,25,26,27,28,29,30,31,32,33,34].

**Figure 1:**
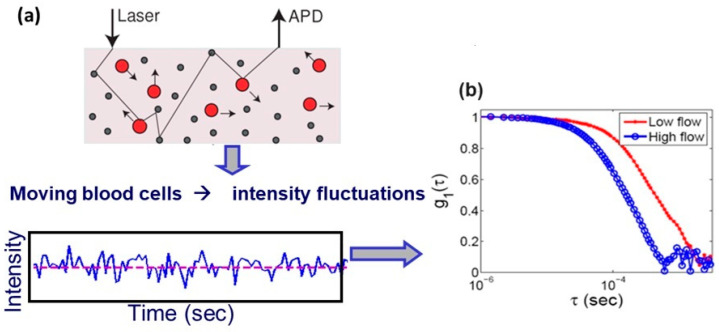
Illustration of the DCS process. (**a**) Multimode laser enters the tissue, and the scattered light is detected by the APD shown as light intensity. (**b**) The autocorrelation of the scattered light. Faster decay in the correlation indicates high flow.

A typical DCS system is composed of an optical component and an electronic component (Figure 1). The optical components consist of a multi-mode source fiber coupled to a high-coherence laser and a single-mode detector fiber coupled to an avalanche photodiode (APD). When the laser fiber illuminates the tissue, the intensity of the scattered light is monitored by the APD through the detector fiber. Each pulse from the electrical output of the APD corresponds to a single captured photon. The movement of scatterers in the tissue, primarily red blood cells, causes fluctuations in the intensity over time that can be quantified with a temporal intensity autocorrelation function. The decay of the correlation is related to the motion of the light scatterers. Fast-moving scatterers cause more rapid changes in the scattered light; therefore, faster decay in the correlation indicates faster motion [1,2,3,4,5,35,36]. A diffusion correlation equation relates the rate of the decay of the correlation to the BFI [1,2,3,4,5]. Therefore, by fitting this model equation to the measured correlation, the BFI can be quantified.

The electronic component of DCS has three major features: counting the number of photons in a predefined time bin, calculating the autocorrelation of the photon counts, and fitting the measured correlation to the diffusion correlation equation to derive the BFI. Correlation and fitting are both computationally intensive tasks. For the correlation calculation, most DCS systems employ either a counter acquisition board that counts output pulses from the detector connected to a computer with a software correlator [37,38,39] or a commercial hardware correlator board that performs both functions [1,2,3,4,5]. Each of these configurations comes with limitations. Software correlators are flexible and can be configured to only calculate physiologically relevant correlations for predetermined delay times, but they require high-performance computers to perform the calculations, which can take a long time to complete especially for the high frame rate of DCS data acquisitions. Moreover, in neuro-intensive care units, clinicians are interested in having real-time BFI as an additional local cerebral health metric as an adjunct to the other standard clinical monitoring parameters such as mean arterial pressure (MAP), heart rate (HR), oxygen saturation (SpO_2_), intracranial pressure (ICP), etc. Commercial hardware correlators are more powerful as they are based on field-programmable gate arrays (FPGAs) that can perform the calculations in real-time. However, they are less flexible and hard for end users to reconfigure. Several custom, open-source FPGA correlator designs have been made that provide much greater flexibility, but they have not yet been applied to DCS applications [40,41,42,43,44,45].

The BFI is obtained by fitting a theoretical diffusion correlation equation with the acquired correlation values. The curve fitting can be performed with a least-squares criterium. The residual sum of squares between the theoretical equation and the acquired correlation is calculated and then minimized using algorithms like the Nelder–Mead method [46]. The fit is nonlinear and computationally intensive. Currently, curve fitting for DCS is mostly performed in software. As a result, these fits are difficult to do in real time and are often carried out in post-processing [44,46]. Therefore, it is difficult to perform real-time DCS measurements, especially with multiple DCS channels running at a fast measurement rate without a high-performance computer. Lin et al. [44] developed a DCS analyzer that successfully implemented the curve fitting algorithm with the Nelder–Mead method on FPGA. The design was made using LabVIEW FPGA development tools (LabVIEW 2018, National Instruments, Austin, TX, USA) and tested on a National Instruments FPGA module. It was shown to be able to achieve a processing speed of less than 1 ms per fit. This demonstrated the potential for offloading DCS data processing to an FPGA and the development of a portable DCS system.

The focus of this work is to develop a single-chip solution for the electronic component of the DCS system. This is the first report of such work in the DCS community. It integrates a photon counter, a hardware correlator, a DCS analyzer, and a system control micro-controller on one FPGA chip using the Very High-Speed Integrated Circuit Hardware Description Language (VHDL). The design is universal and can be implemented on any FPGA chip with sufficient resources. The advantage of our solution is the real-time data processing at a high data rate to reveal the dynamics of blood flow. The DCS system presented here significantly reduces the computational hardware needed for DCS and easily allows for real-time processing at high measurement rates. The system performance will be defined by the processing rate of the DCS analyzer and the overall BFI measurement rate in real-time DCS. Real-time processing has clinical importance, for example, during the longitudinal monitoring of patients at neuro-intensive care units or during interventions where real-time feedback to clinicians can guide decision making.

## 2. Materials and Methods

Our DCS system is composed of several high-level components integrated into our FPGA. First, a photon counting module takes the direct electrical output from the APD to determine the photon counts within a preset time bin. Second, the binned counts are passed to a correlator module to generate the autocorrelation of the light intensity. Finally, a DCS analyzer module performs the curve fitting process to extract the final BFI result. The operation of these modules is controlled by a Microblaze soft microprocessor core (AMD, Santa Clara, CA, USA).

### 2.1. DCS Analyzer Module

The DCS analyzer for our system is based on the architecture of the analyzer previously presented by Lin et al. [44]. We follow the same principle of curve fitting by minimizing the residual sum of squares between the measured autocorrelation and the correlation of the theoretical model. The minimization is performed using the Nelder–Mead method, which is an iterative algorithm for minimization without the calculation of gradients [46]. The basis for this algorithm is performing operations on a simplex to efficiently converge to the solution that minimizes the function. The operations allow the simplex to explore the search space for the minimum of the function through geometric reflections, contractions, expansions, and shrinks. The DCS analyzer used the correlation diffusion equation for semi-infinite diffuse medium model [1,2,3,4,5], where *αD_B_* is equivalent to BFI. The simplified version of the equation can be represented as shown previously by Lin et al. [45]. This leaves two free parameters to fit, the αD_B_ (BFI) and β. Thus, we are working with a two-dimensional space and our simplex is a triangle. The αD_B_ and β values represent the x and y coordinates for each vertex of the triangle.

(1) $$g_{2}=1+\beta {\left(\frac{e^{-r_{1}\sqrt{A+B\alpha D_{B}\tau }}}{r_{1}H}-\frac{e^{-r_{b}\sqrt{A+B\alpha D_{B}\tau }}}{r_{b}H}\right)}^{2}$$

where

$A=3\mu_{s}^{\prime }\mu_{a}$

$B=6{\left(\mu_{s}^{\prime }\right)}^{2}\kappa_{0}^{2}$

$r_{1}=\sqrt{{\left(\frac{1}{\mu_{s}^{\prime }}\right)}^{2}+\rho^{2}}$

$r_{b}=\sqrt{{\left(2z_{b}+\frac{1}{\mu_{s}^{\prime }}\right)}^{2}+\rho^{2}}$

$H=\left(\frac{e^{-r_{1}\sqrt{A}}}{r_{1}}-\frac{e^{-r_{b}\sqrt{A}}}{r_{b}}\right)$

$z_{b}=\frac{2}{\mu_{s}^{,}}\frac{1+R_{eff}}{3\left(1-R_{eff}\right)}$

Equation (1). The semi-infinite solution to the correlation diffusion equation. A, B, H, r_1_, and r_b_ are fixed parameters derived from ${µ}_{s}^{\prime }$, the reduced scattering coefficient; ${µ}_{a}$, the absorption coefficient; $\kappa_{0}$, the wave number; ρ, the source–detector separation; and $R_{eff}$, the effective Fresnel reflection coefficient. The free parameters that are fit by the analyzer are αD_B_ and β.

**Figure 2:**
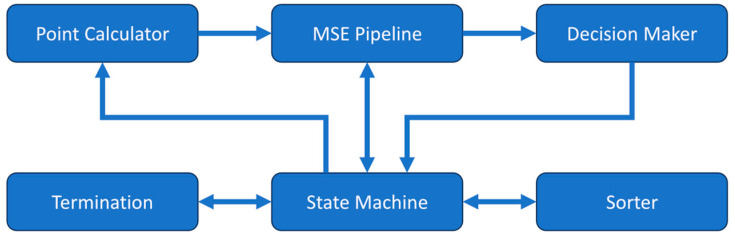
Block diagram of the submodules making up the DCS analyzer module. A central state machine controls the operation and flow of data between the other submodules.

The DCS analyzer uses a central state machine along with several submodules to perform the curve fitting operation with the Nelder–Mead method as shown in Figure 2. The equation constants are stored in registers and the measured correlation along with the associated time delays are stored in memory prior to the fitting process for later use. When starting the analyzer, the state machine takes an initial simplex formed by three pairs of β and αD_B_ then passes them to the mean squared error (MSE) pipeline module. The MSE pipeline takes a β-αD_B_ coordinate pair and calculates the theoretical correlation given by the semi-infinite model in Equation (1). It receives the constants used in the equation from the state machine and then determines the MSE between the calculated theoretical correlation and the measured correlation. The three β-αD_B_ pairs and their corresponding MSE values are then sorted by the sorter module to find the best, good, and worst points, which correspond to the lowest, middle, and highest MSEs, respectively. This initial simplex is then sent to the point calculator module. This module calculates new β-αD_B_ pairs for the reflected, contracted, expanded, and shrunk locations according to the rules defined in the Nelder–Mead method. The β-αD_B_ pairs are then passed in the above order to the MSE pipeline module to determine their MSE values for the decision-maker module to find the best β-αD_B_ pair that should be used to replace the previous worst pair in the simplex.

The decision-maker module determines the best β-αD_B_ choice following the rules outlined by the Nelder–Mead method [46,47]. It may find the β-αD_B_ pair before the MSE values of all the new β-αD_B_ pairs are determined. At this point, the MSE pipeline is cleared so that the next iteration of the Nelder–Mead method can start early. The termination module then checks if convergence has been reached. The conditions for the termination of the search are either the percent difference between the best and worst points in the Nelder–Mead simplex being less than the preset threshold or exceeding the maximum number of allowed iterations of the algorithm. If the convergence is not met, the iteration will start on the newly generated simplex.

### 2.2. Autocorrelator Module

The correlator used for this DCS system is a flexible hardware FPGA correlator we previously described [45]. This correlator was designed to be very flexible, allowing for numerous configurations of the calculated delays and the choice between auto- and cross-correlation. For our application, we configured our correlator to calculate the autocorrelation function. We also use a multi-tau scheme that calculates the correlation for a quasi-logarithmic scale of delays. We made two different correlator delay configurations for use with two experiments. One configuration calculated 80 total delays for use with physiological measurements and liquid phantoms while the other calculated 120 delays for use with solid phantoms to capture the slow decay. Both configurations use a 16-delay first stage followed by the appropriate number of multi-tau stages, each calculating 8 delays. Only one of these correlator configurations is implemented in the FPGA at a time.

### 2.3. Full DCS System Integration

The optical components used for our DCS system are standard for continuous wave (CW) DCS. The light source is from a long coherence length, 785 nm continuous laser (DL785-100, CrystaLaser Inc., Reno, NV, USA) coupled to a 600 µm multi-mode fiber. The 5 µm single-mode detector fiber is coupled to an APD (SPCM-780-12-FC, Waltham, MA, USA).

**Figure 3:**
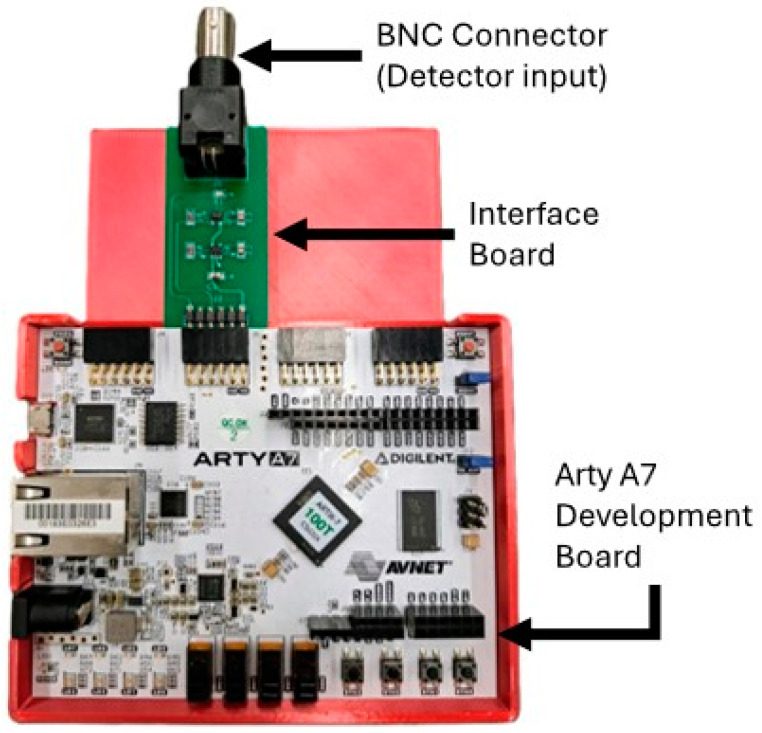
Arty A7 FPGA development board and the custom interface board connected to a Pmod port.

The APD output is a series of approximately 2 V pulses and requires a 50 Ω load. We used the Arty A7-100T FPGA development board (Digilent, Inc., Pullman, WA, USA) for this project. The board hosts a Xilinx Artix-7 FPGA (XC7A100TCSG324-1, AMD, Santa Clara, CA, USA), which has 101 k logic cells, 240 DSP units, and 4860 kbits of block memory. The evaluation board provides Pmod connectors that allow for I/O to the FPGA. Since the I/O pins for the FPGA are configured to use 3.3 V LVCMOS logic levels and do not have the proper 50 Ω impedance, an interface board was built to bring the APD pulse into the FPGA. We built a custom-printed circuit board to provide the interface between the BNC output of the detector and the Pmod port on the FPGA as shown in Figure 3. It provides the necessary termination resistor for the APD output and performs a logic level shift to raise the voltage to the 3.3 V LVCMOS logic level usable by the FPGA.

**Figure 4:**
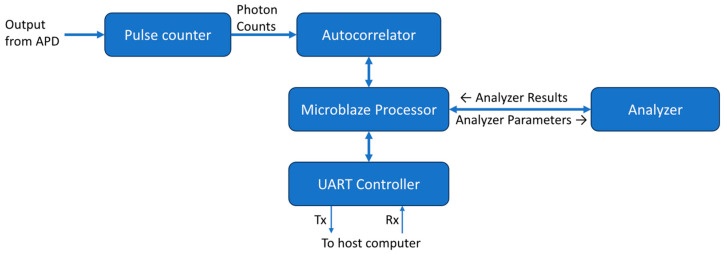
Block diagram showing the major FPGA modules used for the full DCS processing system. The single-headed arrows represent unidirectional data flow while the double-headed arrows represent bidirectional data flow. A Microblaze soft microprocessor core is used to integrate the different components making up the system.

The overall design of the DCS processing system is shown as a block diagram in Figure 4. All the modules run at a frequency of 100 MHz. A MicroBlaze softcore microprocessor Xilinx IP (Xilinx, Inc., San Jose, CA, USA) is used as the system controller. Its primary function is to control the flow of data through the correlator and analyzer. The MicroBlaze IP is configured as a 32-bit processor with a five-stage pipeline, basic floating-point unit, and 32 kB of on-chip block RAM. It also has an interrupt controller supporting fast interrupts to reduce latency during interrupt handling. It has an AXI4-Lite interface available for peripheral connections.

The photon counter module counts pulses from the APD within a time bin of a predetermined duration. The time bin used by our system was 1 µs and is controlled by a dedicated timer. When the timer times out at 1 µs, the module outputs the counter value and resets it for the next 1 µs time bin. The binned counts from the pulse counter module are then passed to the previously described autocorrelator module to calculate the intensity autocorrelation.

The measured correlation from the correlator is written into a dual-port block RAM. One port is used by the correlator to write the correlation and the other is used by the MicroBlaze processor to read from the memory. The flag signal of the last data output from the correlator is used as an interrupt to inform the MicroBlaze processor that new correlation data are ready for processing. The processor performs basic checks on the correlation data. It ensures that the first and last data points of the autocorrelation are within the expected range. If the autocorrelation passes the check, it will be sent to the DCS analyzer along with the necessary correlation model parameters. The analyzer issues an interrupt to inform the processor when curve fitting is complete and that the results are available. The interrupt handler of the processor then retrieves these data.

A UART control module was made for communication between the FPGA and a host computer so the results could be displayed and further analyzed. This module takes the autocorrelation and analyzer output data and sends them over a UART serial interface. An open-source UART module [48] was used to provide an 8-bit-wide streaming interface and make the 3.125 megabaud UART communication port. A 4096-byte deep FIFO was used to buffer data to the UART interface. Our FPGA development board has a UART to USB module that then allows for direct connection to a PC. A Windows GUI application was created for the control of the DCS system and display of the data output. This application allows for the input of the optical absorption coefficient (µ_a_), reduced scattering coefficient (µ_s_′), and the separation between source and detector fibers for a particular measurement. It then uses these values to determine constants used for the DCS analyzer fitting before sending them to the FPGA. The GUI software then plots the autocorrelation and analyzer outputs in real-time as the FPGA generates them and saves the data to a file for further analysis.

### 2.4. System Evaluation and Comparison

Our DCS system was evaluated by comparing its output to a lab-standard reference DCS system widely used by the DCS community, which consists of a 4-channel hardware correlator (Flex01LQ-05, Correlator.com, Bridgewater, NJ, USA) and a computer to perform software curve fitting to determine the BFI in post-processing as we have described in detail previously [22,49,50]. A BNC splitter was used to feed the APD output signal to both the reference system and our FPGA DCS device so that both systems received the same photon pulses with identical optical setups. Several tests were conducted to compare these systems including phantom measurements and cuff ischemia experiments. All these tests used the same source–detector separation of 1 cm.

The first phantom test was with a solid optical tissue phantom made from silicone (µ_s_′ = 17.2 cm^−1^; µ_a_ = 0.23 cm^−1^). Since a solid phantom does not have any motion, we expected there to be near zero measured flow with this phantom and establish the lower limits of these DCS systems. Since this low flow corresponds with a correlation that decays late, we used our 120-channel correlator configuration to fully capture the decay of the correlation. For other experiments, an 80-channel configuration is sufficient and allows for faster fitting.

Liquid phantoms consist of light-scattering particles suspended within a liquid medium. Unlike solid phantoms, liquid phantoms have Brownian motion that can be measured with DCS. By varying the viscosity of the liquid, we can change the rate of this motion. We prepared our base liquid phantom with a 2% dilution of Intralipid (Fresenius Kabi, Uppsala, Sweden) (µ_s_′ = ~20 cm^−1^) with 100 µL/L of India ink (Speedball, Super Black India Ink 385460, Statesville, NC, USA) (µ_a_ = ~0.14 cm^−1^). We also made liquid phantoms with increased viscosities through the addition of methyl cellulose (The Candlemaker’s Store, Methyl Cellulose (Non-FDA) SADMETC, Hamilton, OH, USA). Methyl cellulose concentrations of 0%, 0.0625%, 0.5%, and 1% were chosen to show four distinct flow rates as the flow decreases exponentially with the concentration of methyl cellulose [51]. For these phantom tests, we collected 30 s of data for each phantom at a measurement rate of 1 Hz, i.e., the BFI was computed every second from one second of photon count data.

**Figure 5:**
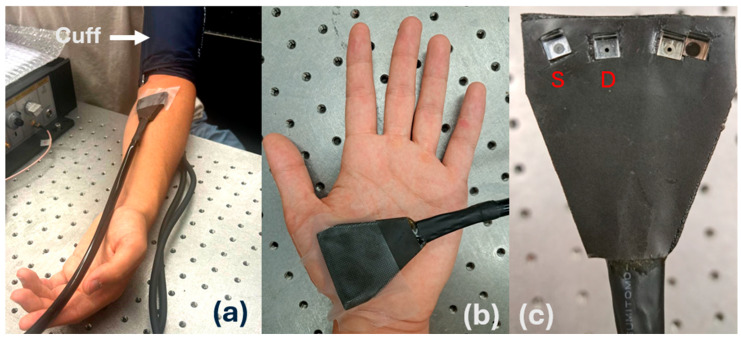
The experimental setup for arm ischemia. (**a**) The blood pressure cuff was placed around the bicep of the arm and the probe was placed on the forearm. (**b**) The experiment was repeated at the subject’s palm. (**c**) The DCS probe used for cuff ischemia experiments. The source (S) and detector (D) have a separation of 1 cm with the other prisms remaining unused for these experiments.

We also performed arm cuff ischemia experiments to show the application of our system for the real-time measurement of muscle blood flow in humans (Figure 5). While the subject was sitting in the full Fowler’s position, a blood pressure cuff was placed around the bicep of the subject’s arm. The subject’s arm was placed on a table to allow for stable measurement and an optical probe was secured to the measurement site. The subject was asked to sit for several minutes to allow for the blood flow to stabilize. A total of 10 s of baseline measurements were taken before inflating the blood pressure cuff up to 200 mmHg to cut off blood flow. The pressure was held for 25 s before releasing it and the measurement lasted until the blood flow returned to baseline. This experiment was performed three times with different measurement rates, one with a measurement rate of 1 Hz at the forearm for both systems, another at the forearm with the FPGA system set to 50 Hz, and the last performed on the palm with the FPGA system set to 50 Hz. We did not use a higher measurement rate for the reference system because its fastest measurement rate is 1 Hz.

## 3. Results

### 3.1. Optical Phantom Tests

To validate the accuracy of our correlator module, we first compared the results from our FPGA correlator module to those from the reference hardware correlator, referred to as the reference here for short. This allows us to see how well the correlations match prior to the curve fitting analysis and see the changes in the correlations with varying flow. For the purposes of display, the 30 correlations collected over each 30-s measurement period were averaged together.

**Figure 6:**
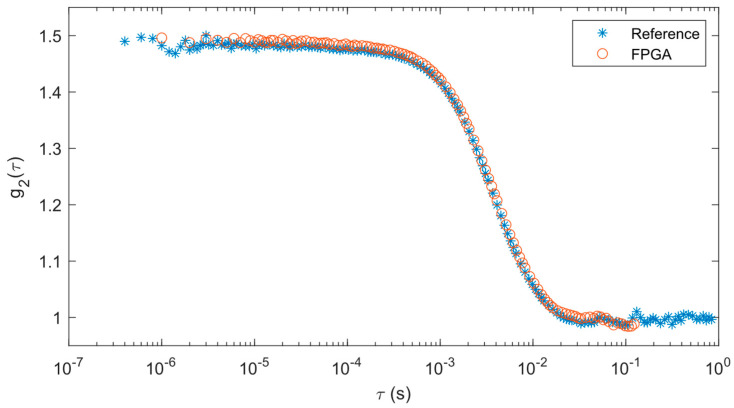
Correlations averaged over 30 s for solid phantom measurements for reference and FPGA correlators.

The correlations from the solid phantom test are shown in Figure 6. Since our FPGA correlator uses a different tau configuration than the reference correlator, the data points between each set of data do not exactly overlap with each other. However, the correlation data from the FPGA correlator matches well with the reference results such that both the correlations decay at the same rate.

The results from the curve fitting analysis provide a quantitative way of comparing these two approaches. The means and standard deviations of the BFIs derived from the curve fitting operations are shown in Table 1. It should be noted that the curve fitting performed with the reference system and FPGA results are slightly different (~11%), most likely due to the differences in these algorithms. While the mean of the FPGA BFI is higher than the reference system, when considering the standard deviations, they are well within range of each other.

**Figure 7:**
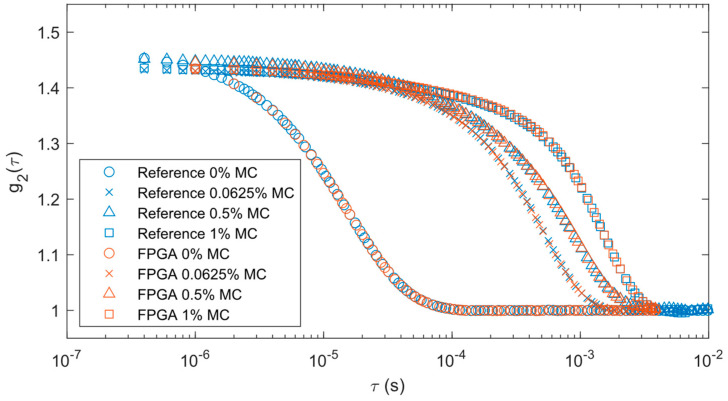
Correlations averaged over 30 s for liquid phantom measurements with varying levels of methyl cellulose (MC) for reference and FPGA correlators.

We processed our liquid phantom results in the same way as the solid phantom. A comparison of the correlations between the two systems can be found in Figure 7. The blue markers represent the output from the reference correlator while the brown markers are for the FPGA data. The different marker shapes represent the varying levels of methyl cellulose. The FPGA correlations remained consistent with the reference system results with each concentration of methyl cellulose. The correlation decay rates were slower as the methyl cellulose increased in concentration because it increased liquid viscosity.

The curve fitting analysis results for the liquid phantoms are shown in Table 2. There is an expected decrease in the mean flow as the concentration of methyl cellulose increases. The standard deviation of the measurements also decreases proportionally with the mean as the concentration increases.

### 3.2. Cuff Ischemia Tests

**Figure 8:**
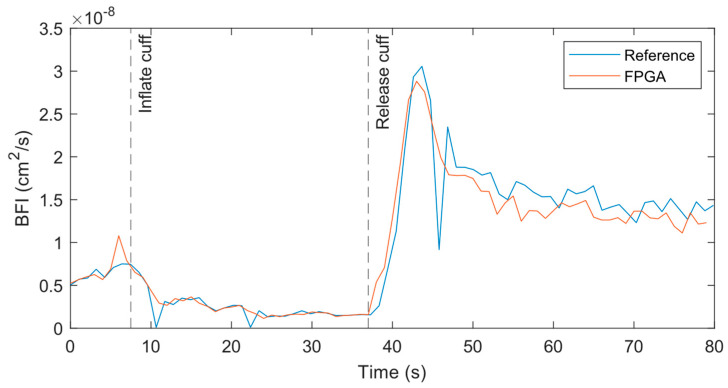
BFI from reference and FPGA in cuff ischemia experiment with both systems measuring at 1 Hz on the forearm with a source–detector separation of 1 cm.

At the 1 Hz measurement rate, the BFI data from both systems match well across the experiment period when performed at the forearm, as shown in Figure 8. At the initial baseline, the flow for both systems is nearly identical with similar variations over time. As the cuff is inflated, the BFI decreases as expected until the cuff is released. At that point, the BFI rises suddenly due to reactive hyperemia. The data from the reference systems has some outliers where the BFI dips or spikes far from surrounding data points. A closer review of the relevant correlations found that poor fits caused these outliers.

**Figure 9:**
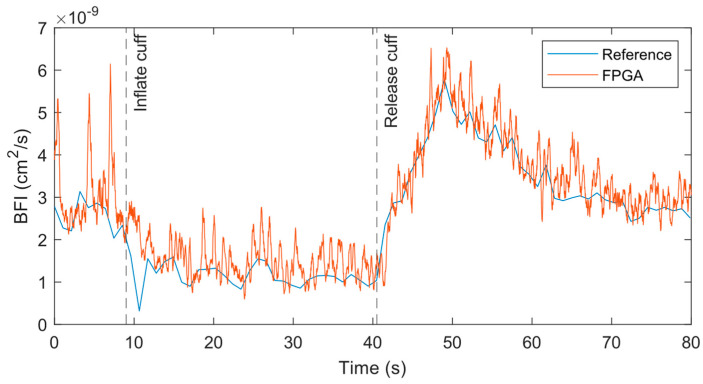
BFI from the reference and FPGA in the cuff ischemia experiment with the reference measuring at 1 Hz and the FPGA at 50 Hz at the forearm with a source–detector separation of 1 cm. A 15-point moving average filter was applied to the FPGA data to remove high-frequency noise.

To resolve the pulsatile flow in the BFI, we increased the measurement rate to 50 Hz for the FPGA system, while the hardware correlator-based reference system could only allow 1 Hz. The original 50 Hz output contains high-frequency noise that makes it difficult to visualize the heartbeat and dicrotic notch. However, with a simple 15-point moving average filter, we could obtain the data shown in Figure 9 indicating a clear pulsing in the BFI at a normal heart rate frequency. A wider, more aggressive filter could have been used, but at the cost of representing less accurately the amplitude of the pulsatile peaks. We are also able to observe the dicrotic notch like the previous work on fast DCS [39]. As with the prior 1 Hz measurement, the general trends in the BFI between the 50 Hz FPGA and 1 Hz reference measurements were nearly identical.

**Figure 10:**
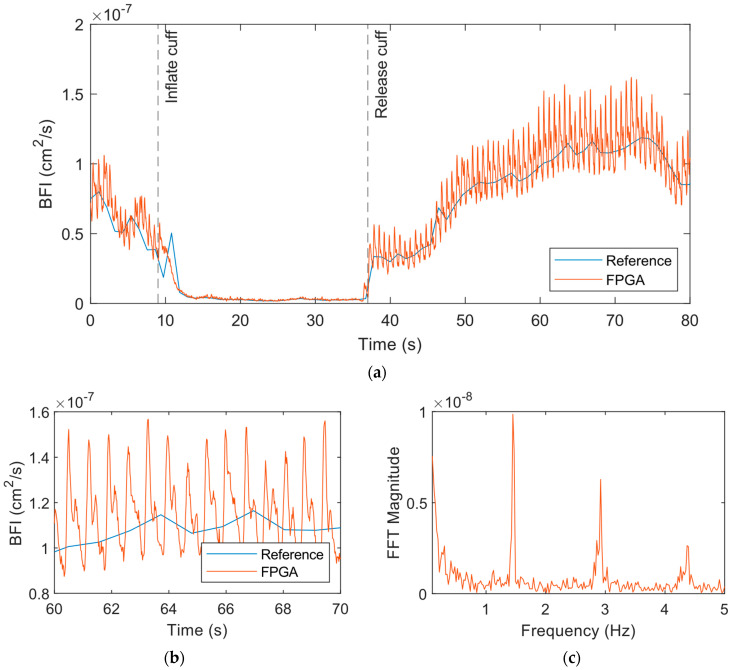
BFI from the reference and FPGA in the cuff ischemia experiment with the reference measuring at 1 Hz and the FPGA at 50 Hz at the palm with a source–detector separation of 1 cm. A moving average filter with a window size of 5 data points was applied to the FPGA data to remove high-frequency noise. (**a**) The full time series from the experiment. (**b**) Zoomed in view of 10 s of the time series data. The pulsatile flow and dicrotic notch are clearly apparent here. (**c**) The frequency spectrum from an FFT of the FPGA BFI data following the release of the cuff. The peak at 1.45 Hz corresponds to the heart rate of the subject.

Next, Figure 10 shows the results from the experiment on the palm. We used a smaller 5-point moving average filter for these data since these data were less noisy than the forearm. The results showed a similar drop in BFI as the cuff was inflated (Figure 10a), but the pulsatile amplitude also decreased significantly during this period. Upon the release of the cuff, the BFI increased and then came to baseline with time. The pulsatile flow quantified by the FPGA system was much more pronounced and the dicrotic notch was clearly apparent (Figure 10b). The frequency spectrum data following the release of the cuff indicated a peak at 1.45 Hz corresponding to the heart rate of the subject (Figure 10c). The other peaks correspond to the harmonics of the heart rate.

## 4. Discussion

The FPGA implementation of a full DCS data processing system on a single chip shows the potential for making DCS more accessible for portable and wearable applications. Our FPGA implementation provides a complete device-on-chip solution for real-time DCS electronics from the direct output of an APD to the final BFI. With the integration of the correlator and analyzer modules on the same FPGA chip, we can achieve fast and accurate DCS analysis at a measurement rate of 50 Hz which is highly suitable for real-time applications. Our FPGA correlator can replace the function of expensive and inflexible hardware correlators with a customizable implementation tailored to specific physiological applications. Our FPGA analyzer can perform the curve fitting operation quickly, usually in approximately 150 µs, which was confirmed by an embedded timer in the FPGA. This easily allows for real-time BFI analysis even at high measurement rates. There is no reliance on a high-performance computer for post-processing or the use of less accurate, simplified fitting procedures in software.

Our design is resource-efficient as it uses only part of the relatively inexpensive and small FPGA on our development board. The whole design uses 15,810 LUTs (25%), 19,224 registers (15%), 15.5 block RAMs (11%), and 123 DSPs (51.25%) on an Artix-7 100T FPGA. The current single-channel implementation of our design shows only a fraction of the potential it has for enabling multi-channel, fast DCS measurements. Due to the immense speed at which our correlator and analyzer operate, we can fit more channels on our FPGA chip. In its current form, our correlator operates at a frequency of 100 MHz and can accept a new data point on every clock cycle. Our experiments used a photon counter binning size of 1 µs for a single channel so a new data point is generated for the correlator at a rate of 1 MHz or once every 100 clock cycles. This leaves the correlator idle for the 99 remaining clock cycles. Instead of idling, we can use these clock cycles to perform correlations for other channels. This would theoretically allow for 100 channels to be correlated using the same correlator hardware. With an approximate average of 150 µs per curve fit with our analyzer module, we can perform over 6000 fittings per second. For a DCS measurement rate of 50 Hz, one analyzer could process the data for up to 120 channels. Although the numbers are theoretical, they demonstrate the potential of our technology for use in a multi-channel, real-time DCS system.

A comparison of the results across our phantom and cuff ischemia experiments showed that our FPGA system was equivalent to the reference DCS system. A direct comparison of the correlations for each system shows matching results. The BFI results coming out of our FPGA DCS analyzer show that our implementation is equivalent to the reference software curve fitting algorithms. The BFI data from the liquid phantom experiments showed that the BFI values from the FPGA system were slightly different than the reference system, possibly due to the small differences between the curve fitting of our FPGA and the software used for the reference system. For example, the FPGA system has 80 correlation values that are used for the curve fitting, while the reference system has 128, of which a segment of the correlation is selected for fitting. However, it should not affect the performance of the DCS system in practice as shown in the cuff ischemia test. Pulsatile flow could be resolved from our forearm cuff ischemia and palm measurements. We used a relatively short source–detector separation of 1 cm for all our experiments, which means we are measuring flow at a shallow depth. The highly vascularized and muscular area of the palm by the base of the thumb has more superficial flow than our forearm location which allows us to resolve much clearer pulsatile flow with the same equipment and signal–noise ratio.

FPGAs have been previously used in DCS systems as the hardware autocorrelator. It has become more effective in applications that involve SPAD arrays for the autocorrelation of multiple channels [52,53,54]. In time-domain DCS systems, FPGAs are used for tagging detection events to derive the autocorrelation [55,56]. The uniqueness of our solution is the inclusion of the DCS analyzer in addition to the hardware correlator on the same FPGA chip. It can output not only the autocorrelation of the photon correlation in real-time, but also the BFI at the rate of 6000 BFIs per second. If the same process is carried out by software, the BFI output rate is limited to the range of 20 BFIs per second [44,53]. Therefore, our DCS solution has the advantage of a fast data output rate with a small device footprint.

Our approach has several limitations. Although the components of our design have flexible configurations and are easy to use, the post-processing software approach still offers a much more flexible solution for DCS research. Our analyzer implementation is fixed to the semi-infinite correlation diffusion equation, and it cannot be changed without major modifications to the FPGA logic. Changing any of the algorithms or models requires much more time than the software code that can be easily updated. However, this can be mitigated by using the dynamic partial reconfiguration technology [57] available for FPGA to update the logic for the relevant module in real-time. Furthermore, a two-layer analytical, Monte Carlo model [14,58,59,60,61,62], or deep learning implementation [63,64,65,66,67,68,69] may be a more accurate approach. Additionally, our DCS analyzer uses the Nelder–Mead method to minimize the error between the measured and theoretical correlation. This method of minimization is much more practical to implement on an FPGA than gradient-based methods, but it can be susceptible to getting stuck at local minimums that generate suboptimal results. We largely eliminate this issue by providing an initial simplex to our analyzer that is in the expected range of the results and has had no observable issues with our methodology.

## 5. Conclusions

We have shown the development of a fully integrated DCS processing system that can perform all the calculations necessary to produce a BFI from the output pulse of the photon detector on a single FPGA chip in real-time. We have validated our FPGA system against an existing lab standard DCS (reference) system and shown comparable results. Our system also has the capability to perform fast DCS measurements and can resolve pulsatile blood flow. Our future work will be to support multiple DCS channels with a shared correlator and analyzer hardware. The development of this system can significantly lower the cost and footprint of the computational components of DCS and pave the way for portable, real-time DCS systems.

## Figures and Tables

**Table 1:** Means and standard deviations of BFIs derived from 30 solid phantom correlations for reference and FPGA systems.

Data Source	Mean ± Standard Deviation (×10^−11^ cm^2^/s)
**Reference**	9.869 ± 2.762
**FPGA**	10.961 ± 2.956

**Table 2:** Means and standard deviations of BFIs derived from 30 liquid phantom correlations each from solutions of varying methyl cellulose concentrations for reference and FPGA systems.

Methyl Cellulose Concentration (%)	Data Source	Mean ± Standard Deviation (×10^−11^ cm^2^/s)
**0.0625**	**Reference**	12.548 ± 2.1360
	**FPGA**	12.009 ± 2.6367
**0.5**	**Reference**	8.2673 ± 1.7543
	**FPGA**	7.6094 ± 1.5856
**1**	**Reference**	4.1740 ± 0.59629
	**FPGA**	3.8712 ± 0.43825

## Data Availability

Data are contained within the article.
